# Aneurysm of right sinus of valsalva revealed by COVID-19 infection

**DOI:** 10.11604/pamj.2021.38.260.27890

**Published:** 2021-03-15

**Authors:** Hammam Rasras, Zalaria Bazid

**Affiliations:** 1 Department of Cardiology, Mohammed VI University Hospital of Oujda, Mohammed First University of Oujda, Oujda, Morocco,; 2 Laboratory of Epidemiology, Clinical Research and Public Health, Faculty of Medicine and Pharmacy, Mohammed the First University of Oujda, Oujda, Morocco

**Keywords:** Aneurysm, sinus of valsalva, COVID-19

## Image in medicine

A 68-year-old man with history of diabetes mellitus, presented to the emergency department for dyspnea with chest pain. On clinical examination, the patient was hypothermic (35.1°), blood pressure at 185/105 mmHg, heart beats at 135n with SpO_2_ at 55%. In the para clinical work up, an inflammatory syndrome (white blood cells: 19,000/mm^3^, C-reactive protein (CRP): 268 mg/l, procalcitonin: 2.9 mg/l, fibrinogen: 8.7 g/l, lactate dehydrogenase: 1352 g/mol with ferretinemia: 2350 mg/l) was found, and because of the pandemic context, a thoracic computed tomography (CT) scan was performed; showing diffuse patchy ground-glass like opacities suggesting COVID-19 pneumonia with severe involvement (75%), a COVID-19 polymerase chain reaction (PCR) test was positive, with D-dimers: 8.40 ùg/l. Transthoracic echocardiogram (TTE) showed: an aneurysm of right sinus of valsalva with diameter at 56 mm, without regurgitation, systolic pulmonary artery pressure at 52 mmHg. Thoracic angio-CT scan showed bilateral pulmonary embolism and dilated aortic sinus at 56 mm. Due to the respiratory instability, the patient was intubated and ventilated. 24 hours later, he presented with an unrecovered cardiac arrest. Sinus of valsalva aneurysms (SOVAs) can be either acquired or congenital. Although there is debate regarding whether congenital or acquired subtypes are more frequent, Sova is a consequence of weakness of the elastic lamina at the junction of the aortic media and the annulus fibrosis. Many studies have reported vascular diseases during COVID-19 infection. This complication can be correlated with two main processes. The first is an endotheliitis, then in later stages of the disease, there is peri/panarteritis.

**Figure 1:**
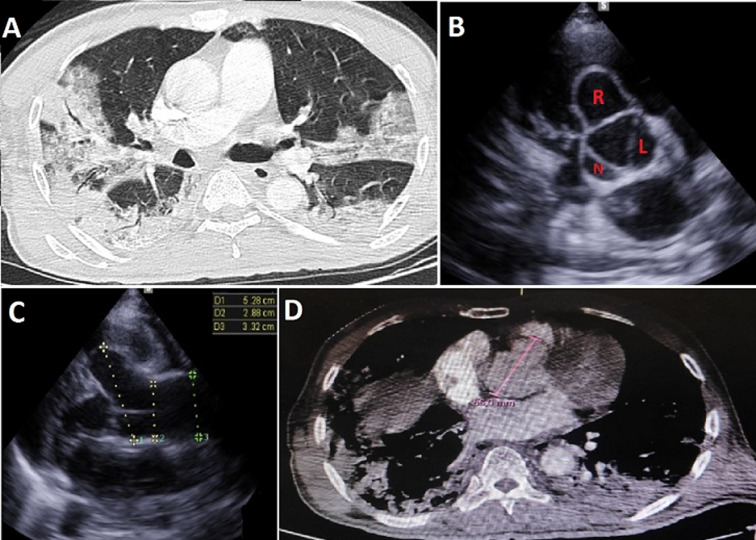
A) thoracic computed tomography scan-axial view showing diffuse patchy ground-glass like opacities suggesting COVID-19 pneumonia with severe involvement (75%); B) transthoracic echocardiogram - small axis parasternal view showing a dilated right sinus of valsalva (R: right sinus, L: left sinus, N: non coronary sinus); C) transthoracic echocardiogram - large axis parasternal view showing a dilated aortic sinus at 52.8 mm; D) thoracic computed tomography scan-axial view showing an aneurysm of right sinus of valsalva with diameter of aortic sinus at 56 mm

